# Occupational Infection Prevention Among Nurses and Laboratory Technicians Amidst Multiple Health Emergencies in Outbreak-Prone Country, D.R. Congo

**DOI:** 10.3390/tropicalmed11010014

**Published:** 2026-01-02

**Authors:** Nlandu Roger Ngatu, Sakiko Kanbara, Christian Wansu-Mapong, Daniel Kuezina Tonduangu, Ngombe Leon-Kabamba, Berthier Nsadi-Fwene, Bertin Mindje-Kolomba, Antoine Tshimpi, Kanae Kanda, Chisako Okai, Hiromi Suzuki, Nzaji Michel-Kabamba, Georges Balenda-Matondo, Nobuyuki Miyatake, Akira Nishiyama, Tomomi Kuwahara, Akihito Harusato

**Affiliations:** 1 Department of Public Health, Faculty of Medicine, Kagawa University, Kagawa 761-0793, Japan; kanda.kanae@kagawa-u.ac.jp (K.K.); harusato.akihito@kagawa-u.ac.jp (A.H.); 2 Global Nursing Section, School of Disaster Nursing, Kobe City College of Nursing, Kobe 651-2103, Japan; s-kanbara@kobe-ccn.ac.jp; 3 Faculty of Dental Medicine, University de Kinshasa, Kinshasa P.O. Box 152, Democratic Republic of the Congo; wansumapong1@yahoo.fr; 4 Department of Internal Medicine, University of Kinshasa, Kinshasa P.O. Box 127, Democratic Republic of the Congo; danieltonduangu@yahoo.fr (D.K.T.); antshimpi@aol.com (A.T.); 5 Faculty of Medicine, University of Kamina, Kamina P.O. Box 279, Democratic Republic of the Congo; leonkab@hotmail.com (N.L.-K.); mindjekolomba@gmail.com (B.M.-K.); michelnzaji@yahoo.fr (N.M.-K.); 6 Department of Surgery, Faculty of Medicine, University of Kinshasa, Kinshasa P.O. Box 127, Democratic Republic of the Congo; berthiernsadif@yahoo.fr; 7 Department of Hygiene, Faculty of Medicine, Kagawa University, Kagawa 761-0793, Japan; okai@kusw.ac.jp (C.O.); suzuki.hiromi@kagawa-u.ac.jp (H.S.); miyatake.nobuyuki@kagawa-u.ac.jp (N.M.); 8 Surgery Unit, Louis-Pasteur Medical Center, Pretoria 0002, South Africa; gbalenda@yahoo.co.uk; 9 Department of Medical Pharmacology, Faculty of Medicine, Kagawa University, Kagawa 761-0793, Japan; nishiyama.akira@kagawa-u.ac.jp; 10 Department of Medical Microbiology, Faculty of Medicine, Kagawa University, Kagawa 761-0793, Japan; kuwahara.tomomi@kagawa-u.ac.jp

**Keywords:** healthcare worker, infection prevention, occupational safety, universal precautions

## Abstract

Millions of healthcare workers experience percutaneous exposure to bloodborne communicable infectious disease pathogens annually, with the risk of contracting occupationally acquired infections. In this study, we aimed to assess the status of occupational safety and outbreak preparedness in Congolese nurses and laboratory technicians in Kongo central and the Katanga area, amidst multiple ongoing public health emergencies in the Democratic Republic of the Congo (DRC). This was a multicenter analytical cross-sectional study conducted in five referral hospitals located in Kongo central province and the Katanga area between 2019 and 2020 amidst Ebola, Yellow fever, Cholera and Chikungunya outbreaks. Participants were adult A0 grade nurses, A1 nurses, A2 nurses and medical laboratory technicians (N = 493). They answered a structured, self-administered questionnaire related to hospital hygiene and standard precautions for occupational infection prevention. The majority of the respondents were females (53.6%), and 30.1% of them have never participated in a training session on hospital infection prevention during their career. The proportions of those who have been immunized against hepatitis B virus (HBV) was markedly low, at 16.5%. Of the respondents, 75.3% have been using safety-engineered medical devices (SEDs), whereas 93.5% consistently disinfected medical devices after use. Moreover, 78% of the respondents used gloves during medical procedures and 92.2% wore masks consistently. A large majority of the respondents, 82.9%, have been recapping the needles after use. Regarding participation in outbreak response, 24.5% and 12.2% of the respondents were Chikungunya and Cholera epidemic responders, respectively; 1.8% have served in Ebola outbreak sites. The proportion of the respondents who sustained at least one percutaneous injury by needlestick or sharp device, blood/body fluid splash or both in the previous 12-month period was high, 89.3% (41.8% for injury, 59.2% for BBF event), and most of them (73%) reported over 11 events. Compared to laboratory technicians, nurses had higher odds for sustaining percutaneous injury and BBF events [OR = 1.38 (0.16); *p* < 0.01], whereas respondents with longer working experience were less likely to sustain those events [OR = 0.47 (0.11); *p* < 0.001]. Findings from this study suggest that Congolese nurses and laboratory technicians experience a high frequency of injury and BBF events at work, and remain at high risk for occupationally acquired infection. There is a need for periodic capacity-building training for the healthcare workforce to improve infection prevention in health settings, the provision of sufficient and appropriate PPE and SEDs, post-exposure follow-up and keeping records of occupational injuries in hospitals in Congolese healthcare settings.

## 1. Introduction

Globally, healthcare-associated communicable infectious diseases affect 100 million patients and 35 million healthcare workers (HCWs) annually mainly due to poor adherence to standard precautions for infection prevention. Such infections represent a serious issue given their high incidence not only among members of the healthcare workforce in health settings but also among patients [1,2,3]. There are approximately 3 million HCWs experiencing percutaneous exposure to bloodborne viral agents annually, such as hepatitis B virus (HBV), hepatitis C virus (HCV) and human immunodeficiency virus (HIV), resulting in psychological stress-associated health outcomes and socioeconomic loss [1,3,4,5]. Accumulated evidence suggests that healthcare settings often play an important role in the spread of infection and epidemic amplification [6]. This is true particularly for countries in the WHO Africa region. Preparing for epidemic threats is a dynamic state requiring periodic capacity-building of HCWs and compliance with infection prevention and control (IPC) measures [7], which require HCWs to consistently adhere to standardized practices such as hand hygiene, needlestick and sharp injury prevention, the use of personal protective equipment (PPE), environmental cleaning and medical waste management [8]. Reports from international health agencies suggest that WHO Africa remains one the most affected regions in terms of emerging epidemics of communicable infectious diseases in the world [9].

In the Democratic Republic of the Congo (DRC), outbreaks of infectious diseases caused by highly transmissible pathogens have spread to several provinces of this well-known epidemic-prone country. Between 2018 and 2020, the DRC has faced major public health emergencies, with Ebola virus disease (EVD), Yellow fever, Measles, Cholera and Chikungunya outbreaks occurring concurrently. Regarding the spread of EVD in the DRC, it has been reported that health settings in affected areas play a significant role in outbreak amplification. When a hospital infection occurs, it is susceptible to serve as the source of pathogen transmission to hospital personnel, inpatients and outpatients who might propagate the infection to other areas [10,11,12,13].

Furthermore, insufficient number of trained frontline HCWs, limited resources and inadequately equipped wards and the exhausted health system further increase the risk of outbreak occurrence [13]. Previous studies that explored hospital hygiene status have shown serious failings that might be associated with the high incidence of occupational infection among HCWs, particularly due to lack of compliance to occupational safety guidelines [14,15,16,17]. Additionally, our previous work and investigations by other researchers have shown a high frequency of occupational percutaneous injury and blood and other body fluid (BBF) splash events in Congolese health settings. The most prevalent factors that expose HCWs to such events include, among others, the absence of periodic training on infection prevention, limited provision of personal protective medical equipment (PPE) and a lack of safety-engineered medical device (SED) usage [15,18,19]. These factors increase the risk of infection not only among healthcare personnel but also among healthcare patients.

Furthermore, unprepared HCWs—nurses in particular—who are frontline healthcare service providers, combined with unsatisfactory working conditions, are likely to increase the risk of outbreak of communicable infectious diseases in the DRC. The present study aimed to assess occupational safety status among Congolese nurses and medical laboratory technicians in Kongo central province and the Katanga area. We hypothesized that at least 50% of the participants would have sustained at least one percutaneous injury, BBF splash event or both in the previous 12 months preceding the implementation of this study.

## 2. Materials and Methods

### 2.1. Study Design, Participants and Sample Size Estimation

This was a multicenter analytical cross-sectional study conducted in five referral health settings, including two university hospitals, between 2019 and 2020, amidst Ebola, Yellow fever, Cholera and Chikungunya outbreaks. The study sites are located in two provinces of the DRC: Kongo central in the western area and Haut-Katanga in the southern area. They are among areas not affected by the longstanding eastern Congo armed conflict, involving regular armies of two neighboring countries, Rwanda and Uganda.

The study population comprised nurses and medical laboratory technicians working full-time at each of the selected health settings. The single proportion formula was used to calculate the sample size of this study, with the assumption that 50% of participants comply with consistent use of personal protective medical equipment (PPE), as we previously reported [20]. Thus, the expected sample size was 158 participants per province, for a total of 316 nurses and laboratory technicians for both Kongo central and Haut-Katanga provinces. In total, 496 nurses and medical laboratory technicians from selected healthcare settings were enrolled; they were invited to participate in this study and return the survey sheets within a week. All lab technicians completed the survey, whereas 3 nurses who could not complete it on time were excluded. Thus, the final sample size was 493. Each participant was given an individually identifiable code used only for research purposes.

### 2.2. Selection Criteria

Recruitment of the participants was carried out through direct contact by at their respective unit or department by hospital staff who have served as survey facilitators, based on the following criteria: being 18 years old or older, working full-time as a nurse or laboratory technician at the selected health setting and not having part-time work in any other health setting. Each participant was given an individually identifiable unique number (code) that was solely used for research purposes.

### 2.3. Survey Questionnaire and Data Collection

A structured, self-administered questionnaire related to standard or universal precautions for infection prevention was used (Supplementary File). It was divided into five sections:

(1) Sociodemographics; (2) training and knowledge on Universal or Standard Precautions (UP); (3) exposure to accidental injury and skin contact with blood or other body fluid (BBF) in the hospital or health setting; (4) compliance to preventive measures for occupationally acquired infection, including consistent use of PPE (gloves, mask) during risky medical procedures, and the use of SEDs such as self-recapping needles, catheters and syringes; and (5) vaccination (anti-HBV, anti-tetanus) and compliance with post-exposure measures and mental health impact of occupational injury or BBF events.

In this study, primary outcome variables were the occupational percutaneous injury caused by needlestick or sharp medical devices, and skin exposure to blood or other body fluid (BBF) events. The secondary outcomes were the frequencies of post-exposure seropositive test results for HBV, HCV and HIV; sociodemographic and occupational characteristics of the respondents were considered as the covariates.

### 2.4. Ethical Considerations

The present study used pseudonymous data collected from nurses (all categories from A0 to A2 grades) and laboratory technicians who voluntarily agreed to participate in the survey. Each questionnaire sheet had a serial number that was used during data transcription into a prepared excel file. A statement on informed consent was provided in the questionnaire. Thus, only workers who agreed with the statement answered the questions. The survey was part of the “Congo Communicable Diseases Research Project” that was approved by the Ethics committee of University of Lubumbashi School of Public Health, DRC (Approval number: UNILU/CEM/070/2016).

### 2.5. Data Analysis

Categorical variables are expressed as proportions or percentages. For each item of the survey questionnaire, descriptive statistical tests were employed. Adherence to the standard precautions suggests that healthcare providers should execute the preventive measures at 100%; otherwise, it would be considered a failure or lack of adherence to the Universal Precautions. Regarding questions related to the professional practices or medical procedures, a respondent was attributed 100% or 0% if they performed or did not. 

For comparison of the outcomes by categories of the respondents, a Chi-squared test or Fisher’s exact test was performed where appropriate. Multivariate logistic regression models were constructed, with adjustment for age and gender, to identify the predictors of outcome variables (accidental injury, BBF event, viral infection seropositivity) for which *p* values were <0.25 in the univariate analysis. Data analysis was performed with the use of STATA software version 18 (StataCorp LLC, 4905 Lakeway Drive, College Station, TX, USA). The significance level was set at a *p* value of less than 0.05.

## 3. Results

### 3.1. Sociodemographic and Occupational Characteristics of the Respondents

Table 1 shows the characteristics of the study participants. Of the 493 HCWs who were included in this study (participation rate: 99%), there were 93 medical laboratory workers, 18.8%, whereas the majority of the respondents were nurses, 400 (81.13%).

There were more female workers, 53.6% (300/493), as compared with their male counterparts. The majority of the respondents were middle-aged workers (31–40 years; 33.8%) and A1 grade nurses (41.6%), and worked in the internal medicine departments (21.9%) of the participating healthcare settings. A greater proportion of the respondents have been working for 6–10 years (32.4%) either as nurses or medical laboratory technicians, followed by those who have been working for 16 years or more (28.0%).

### 3.2. Awareness and Participation in Outbreak Response, Frequency of Percutaneous Injury and Blood/Body Fluid Splash Events, Post-Exposure Measures, Viral Hepatitis and HIV Testing and Mental Health Impact of Occupational Exposure to the Risk of Bloodborne Infection

**Figure 1:**
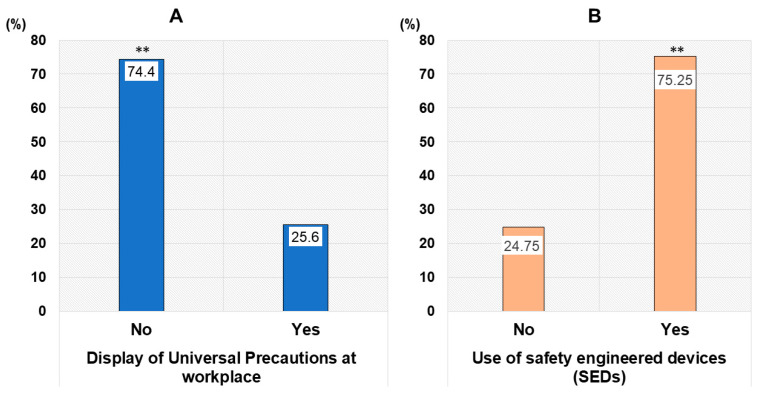
Occupational infection awareness and use of safety engineered medical device (**A**,**B**). Legend: **, *p* value less than 0.01.

A total of 16.5% of the respondents had been immunized against HBV. Displaying posters at the workplace or unit in the hospital that remind healthcare providers of the safety guidelines related to standard precautions for infection prevention is a common practice. The majority of the survey responders, 74.4% (vs. 25.6%), had no such posters displayed at their workplaces (Figure 1A). Higher proportions of the respondents used safety-engineered medical devices (SEDs; 75.25%) consistently and disinfected medical materials and devices after use (93.51%) (Figure 1B). On the other hand, 30.1% (vs. 69.9%) had never participated in training sessions related to occupational safety and standard precautions on infection prevention at any point during their career. Over 78% of the respondents used gloves consistently and the proportion of those wearing masks consistently was high, at 92.2%. Regarding participation in outbreak response within the country, 24.5% and 12.2% of the respondents have worked as Cholera and Chikungunya outbreak responders, respectively, whereas only 1.8% have served as Ebola outbreak responders. A large majority of the respondents (82.93%) have been recapping needles after medical procedures.

**Figure 2:**
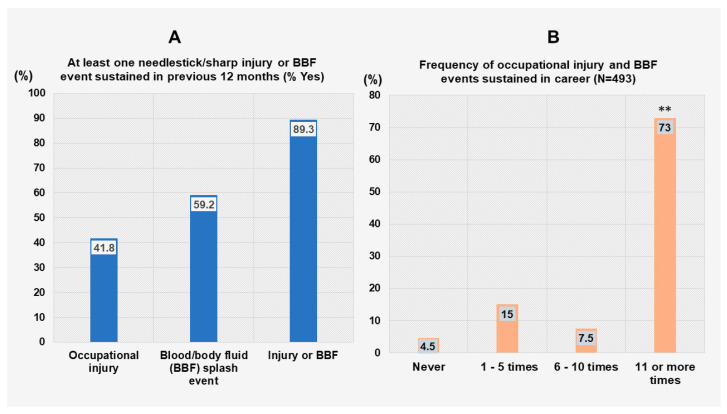
Frequency of injury and blood and body fluid (BBF) splash events occurrence (**A**,**B**). Legend: **, *p* value less than 0.01 (proportion of respondents who sustained 11 or more occupational injury/BBF events versus others).

The occurrence of accidental injury with sharp and other medical devices and skin contact with contaminated body fluids are factors that increase the risk of hospital infection not only for healthcare providers, but also service users who visit the hospitals. This study showed a much higher proportion of the respondents, 89.3%, who have sustained at least one injury, BBF event or both (41.8% for injury, 59.2% for BBF event) in the previous 12 months that preceded this survey (Figure 2A,B). Regarding medical procedures involved in injury or BBF occurrence, injection accounted for 6.55% of the events, followed by blood transfusion, 4.79%, and surgical intervention, 4.79%.

**Figure 3:**
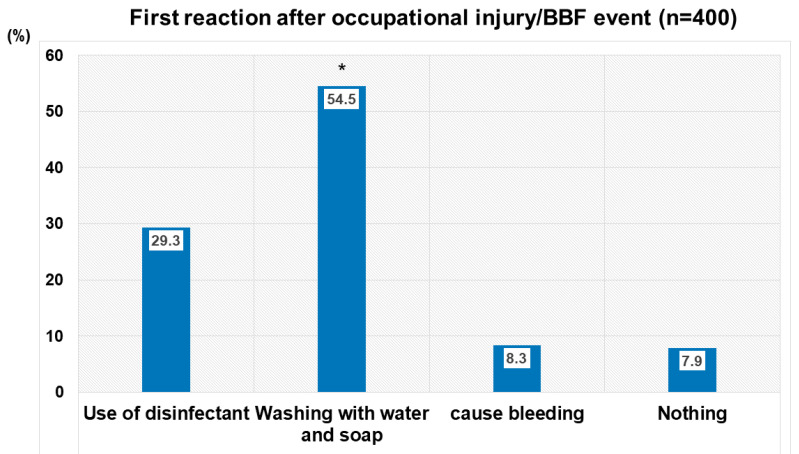
Post-exposure measures applied after occupational injury or blood/body fluid (BBF) splash event. Legend: *, *p* value less than 0.05 (proportion of respondents who washed exposed body area with water/soap as first measure post-injury/blood and body fluid (BBF) splash event versus others).

Most of those respondents reported multiple events occurring within a 12-month period, with 73% having sustained over 11 events, as shown in Figure 2B. Of the respondents who sustained an injury or BBF event at work, approximately half of them (54.5%) reported washing the exposed body area, whereas only 29.3% of them used a disinfectant or caused the exposed site to bleed (8.3%) (Figure 3). Regarding the impact of injury/BBF events on the mental health of HCWs, A0 grade nurses and medical laboratory technicians were the most mentally affected responders by the occurrence of those events, at 82.93% and 84.44%, respectively, followed by A1 grade nurses, at 77.36%.

Overall, 51% of the responders were tested for HIV, and the seropositivity rate was 2.5%. However, the proportions of the respondents who have undergone VHB and VHC testing was markedly low, at 16.5% and 15.3%, respectively; and the rates of positive tests were 6.1% and 9.8%, respectively.

### 3.3. Predictors of the Outcomes by Multivariate Logistic Regression Analysis

Table 2 shows the result of the multivariate logistic regression with adjustment for age and gender. It was observed that the frequency of work-related injury and BBF events occurring in the career of the respondents was associated with gender [OR = 0.40 (0.15), 95%CI: 0.25–0.64; *p* < 0.001], suggesting that women were less likely to sustain occupational injury or BBF splash events; it was also associated with the working years [OR = 0.47 (0.11), 95%CI: 0.32–0.68; *p* < 0.001], with HCWs who have worked longer sustaining less injury and BBF events than those with fewer working years.

Furthermore, the frequency of occupational injury and BBF events was positively associated with occupation [OR = 1.38 (0.16), 95%CI: 1.09–1.74; *p* < 0.01], with nurses being more likely to sustain more occupational percutaneous injury/BBF than medical laboratory workers. Regarding the association between occupational injury/BBF occurrence and viral infection test result, HIV seropositivity was inversely associated with gender [OR = 0.49 (0.16), 95% CI: 0.25–0.94; *p* < 0.005], suggesting that women who sustained injury or BBF events at their workplace were less likely to have a positive HIV serological test (Table 2).

## 4. Discussion

In this study, we explored the status of preparedness and the magnitude of percutaneous exposure to the risk of infection as well as the rate of self-reported positive test for viral infections among different categories of Congolese nurses and medical laboratory workers from five referral health settings. It was observed that, in each category of HCWs, over 80% of the respondents were not immunized against hepatitis B virus. Though high proportions of the participants used SEDs and disinfected medical materials and devices after their utilization, it can be considered that both nurses and medical laboratory workers failed to adhere to the standard precautions given that not all of them participated in training sessions on occupational infection prevention, consistently disinfected medical devices and consistently used disinfectant or running water to wash the exposed body surface after sustaining a percutaneous injury or BBF splash event. This study also revealed a number of striking findings, including the high frequency of self-reported occupational injury/BBF events occurring in the previous 12-month period (89.3%), with 73% of respondents having sustained 11 or more events in their career, and the low coverage of post-exposure testing for common viral hepatitis agents and HIV (15–50%).

Central Africa is one of the regions most affected by communicable infectious diseases. It is well established that the occurrence and the impact of outbreaks of infectious diseases can be reduced by implementing efficient preventive measures and preparedness of the healthcare workplace, more particularly in high-risk areas, and rapid response actions whenever cases of communicable infection arise [21]. Previous studies conducted in Africa and Asia also showed poor adherence to infection prevention guidelines in countries such as Lesotho, Ethiopia, Nigeria, Ghana and Bangladesh [22,23,24,25,26,27,28,29].

In our study, the proportion of the respondents who sustained at least either one percutaneous injury or BBF event or both in the previous 12 months was abnormally high, at 89%. The high frequency of occupational injury among Congolese HCWs is due, at least partially, to needle recapping. In this study, 82.9% of the respondents have been recapping needles after use, which is an unsafe practice that is contrary to the standard precautions. Recent studies conducted in some African countries showed lower rates of needlestick and sharp devices related occupational injuries among healthcare providers. An Ethiopian study that included 196 health professionals found approximately 19% of the participants sustaining such injuries [30]. In South Africa, Laher and McDowal [31] reported a proportion of 26.3% of care providers who have sustained occupational injury caused by needlesticks within a 12-month period in a study that included prehospital emergency medical service personnel.

Furthermore, male respondents, nurses and the respondents with shorter working experience had higher odds for sustaining occupational injury by needlestick, sharp devices or BBF events, whereas male respondents had higher odds for having positive post-exposure HIV test result. We also observed a high proportion (78%) of the respondents who felt anxious or nervous in the aftermath of percutaneous injury or BBF occurrence. A study conducted in Lao PDR showed high score of anxiety and perceived psychological distress among healthcare providers who experienced needlestick and sharp injuries in the previous 6 months [32]. This illustrates the importance of strict adherence to infection prevention and control guidelines, as well as the reinforcement of related policies and the provision of psychological support to HCWs immediately after BBF exposure or occupational injury.

## 5. Strengths and Limitations

The present study is the first investigation to explore the status of occupational safety, occupationally acquired infection, and compare the infection risk between categories of a relatively large sample of Congolese nurses and laboratory technicians. It highlights the poor work safety and high risk for occupational infection in these categories of HCWs in the Democratic Republic of the Congo. Nonetheless, findings in this work were derived from self-reporting of infection rates at work practices, and not from laboratory records and direct observations of practices. In addition, there is limitation inherent to the cross-sectional design of this study, and there is a possibility for recall bias in regard to the exact frequency of injury or BBF events.

## 6. Conclusions

In conclusion, findings from this study, which is the first to explore compliance to standard precautions and the magnitude of occupational injury and BBF in different categories of nursing staff and medical laboratory workers serving in the Democratic Republic of the Congo, suggest that HCWs remain at high risk of occupationally acquired infection and spread of outbreak of infectious diseases. There is a necessity for periodic training for capacity-building purposes related to infection prevention and control, the provision of sufficient and appropriate PPE and SEDs and post-exposure follow-up while keeping records of occupational injuries occurring in hospitals among members of the healthcare workforce.

## Figures and Tables

**Table 1:** Sociodemographic and occupational characteristics of survey respondents.

Characteristics of the Respondents	N (%)
Gender	
F M	300 (53.6) 193 (46.4)
Age (mean; SD)	34 (3.1)
Age category	
18–30 31–40 41–50 51–60 61 or older	132 (26.8) 167 (33.8) 96 (19.4) 70 (14.2) 28 (5.7)
Occupation	
A2 nurse A1 nurse A0 nurse Laboratory technician	119 (24.1) 205 (41.6) 76 (15.4) 93 (18.8)
Department/unit	
Surgery Operation unit Internal med. Obstetrics/Gynecology Anesthesiology Medical laboratory Others	74 (15.0) 19 (3.8) 108 (22.0) 93 (18.8) 13 (2.6) 93 (18.8) 93 (18.8)
Working years	
1–5 6–10 11–15 16 or more	131 (26.5) 160 (32.4) 64 (12.9) 138 (28.0)

**Table 2:** Predictors of events that increase risk of hospital infection.

Predictor	OR (SE)	Z	95% CI	*p*
*Frequency of occupational injury and BBF events during career*				
Age	1.11 (0.12)	1.04	0.91–1.37	0.299
Gender (female vs. male)	0.40 (0.15)	−3.82	0.25–0.64	**0.000**
Occupation (nurse vs. lab technician)	1.38 (0.16)	2.73	1.09–1.74	**0.006**
Working years (longer vs. shorter)	0.47 (0.11)	−3.86	0.32–0.68	**0.000**
*HBV seropositivity rate*				
Age	0.93 (0.23)	−0.27	0.58–1.51	0.385
Gender	0.62 (0.35)	−0.85	0.20–1.88	0.395
Occupation	0.52 (0.24)	−1.44	0.22–1.26	0.150
*HCV seropositivity rate*				
Age	1.06 (0.02)	1.01	0.81–1.10	0.372
Gender	0.47 (0.28)	−1.24	0.15–1.55	0.215
Occupation	0.57 (0.24)	−1.35	0.26–1.29	0.178
*HIV seropositivity rate*				
Age	0.91 (0.13)	−0.63	0.68–1.21	0.530
Gender (female vs. male)	0.49 (0.16)	−2.12	0.25–0.94	**0.034**
Occupation	0.58 (0.23)	−1.32	0.26–1.29	0.186

**Notes:** OR, odds ration; SE, standard error; HBV, hepatitis B virus; HCV, hepatitis C virus.

## Data Availability

The study data can be accessed upon request to the corresponding author (N.R.N.).

## Supplementary Materials

The following supporting information can be downloaded at: https://www.mdpi.com/article/10.3390/tropicalmed11010014/s1, a survey questionnaire.

## References

[1] ILO/WHO Joint ILO/WHO Guidelines on Health Services and HIV/AIDS. International Labour Organization and World Health Organization, 2005. https://www.ilo.org/sites/default/files/wcmsp5/groups/public/@ed_protect/@protrav/@ilo_aids/documents/publication/wcms_116240.pdf.

[2] Michel-Kabamba N., Ngatu N.R., Leon-Kabamba N., Katumbo-Mukemo A., Mukuku O., Ngoyi-Mukonkole J., Ngoie-Mwamba G., Kilolo-Ngoie E., Bwana-Kangulu I., Kafusthi-Mukemo D. (2021). Occupational COVID-19 prevention among Congolese healthcare workers: Knowledge, practices, PPE compliance and safety imperatives. Trop. Med. Infect. Dis..

[3] Abadiga M., Mosisa G., Abate Y. (2020). Magnitude of needlestick and sharp injury and its associated factors among nurses working at health institutions in Western Ethiopia, 2020. Risk Manag. Healthc. Policy.

[4] Lee J.H., Cho J.H., Kim Y.J., Im S.H., Jang E.S., Kim J.-W., Kim H.B., Jeong S.-H. (2017). Occupational blood exposures in health care workers: Incidence, characteristics, and transmission of bloodborne pathogens in South Korea. BMC Public Health.

[5] Babore G.O., Eyesu Y., Mengistu D., Foga S., Heliso A.Z., Ashine T.M. (2024). Adherence to infection prevention practice standard protocol and associated factors among healthcare workers. Glob. J. Qual. Saf. Healthc..

[6] Knight G.M., Pham T.M., Stimson J., Funk S., Jafari Y., Pople D., Evans S., Yin M., Brown C.S., Bhattacharya A. (2022). The contribution of hospital-acquired infections to the COVID-19 epidemic in England in the first half of 2020. BMC Infect. Dis..

[7] Lee V.J., Aguilera X., Heymann D., Wilder-Smith A. (2019). Lancet Infectious Disease Commission. Lancet Infect. Dis..

[8] Cen Y., Lao C., Li Z., Zhao H., Wang T., Fan C., Liu B., Zhao Z., Zou Y., Lin G. (2025). Association between infection prevention and control safety culture and healthcare workers’ compliance with infection control measures: A cross-sectional study. Front. Public Health.

[9] Mboussou F., Ndumbi P., Ngom R., Kassamali Z., Ogundiran O., Van Beek J., Williams G., Okot C., Hamblion E.L., Impouma B. (2019). Infectious disease outbreaks in the African region: Overview of events reported to the World Health Organization in 2018. Epidemiol. Infect..

[10] Nachega J.B., Mbala-Kingebeni P., Otshudiema J., Zumla A., Muyembe-Tamfun J.J. (2021). The colliding epidemics of COVID-19, Ebola and measles in the Democratic Republic of the Congo. Lancet Glob. Health.

[11] Ilunga-Kalenga O., Moeti M., Sparrow A., Nguyen V.K., Lucey D., Ghebreyesus T.A. (2019). The ongoing Ebola epidemic in the Democratic Republic of Congo, 2018–2020. N. Engl. J. Med..

[12] Muzembo B.A., Ntontolo N.P., Ngatu R.N., Khatiwada J., Ngombe K.L., Numbi O.L., Nzaji K.M., Maotela K.J., Ngoyi M.J., Suzuki T. (2020). Local perspectives on Ebola during its tenth outbreak in DR Congo: A nationwide qualitative study. PLoS ONE.

[13] Kasereka M.C., Hawkes M.T. (2019). The cat that kills people: Community beliefs about Ebola origins and implications for disease control in Eastern Democratic Republic of the Congo. Pathog. Glob. Health.

[14] Kinghton S.C., Richmond M., Zabarsky T., Dolansky M., Rai H., Donskey C.J. (2020). Patients’ capacity, opportunity, motivation, and perception of inpatient and hygiene. Am. J. Infect. Control..

[15] Ngatu R.N., Phillips E.K., Wembonyama O.S., Yoshikawa T., Jagger J., Suganuma N. (2012). Practice of universal precautions and risk of occupational blood-borne viral infection among Congolese healthcare workers. Am. J. Infect. Control..

[16] Ngatu R.N., Kayembe N.J.M., Phillips E.K., Okech-Ojony J., Patou-Musumari M., Gaspard-Kibukusa M., Madone-Mandina N., Godefroid-Mayala M., Mutaawe L., Manzengo C. (2019). Epidemiology of ebolavirus disease (EVD) and occupational EVD in healthcare workers in sub-Saharan Africa: Necessity for strengthened public health preparedness. J. Epidemiol..

[17] Aruna A., Mbala P., Minikulu L., Mukadi D., Bulemfu D., Edidi F., Bulabula J., Tshapenda G., Nsio J., Kitenge R. (2019). Ebola virus disease outbreak—Democratic Republic of the Congo, August 2018–November 2019. Mrb. Mrtal. Wkly. Rep..

[18] Shindano T.A., Horsmans Y. (2024). Low level of awareness and prevention of hepatitis B among Congolese healthcare workers: Urgent need for policy implementation. Front. Public Health.

[19] Doshi R.H., Hoff N.A., Bratcher A., Mukadi P., Gadoth A., Nicholson B.P., Williams R., Mukadi D., Mossoko M., Wasiswa J. (2020). Risk factors for Ebola Exposure in Health Care Workers in Boende, Tshuapa Province, Democratic Republic of the Congo. J. Infect. Dis..

[20] Senbato F.R., Wolde D., Belina M., Kotiso K.S., Medhin G., Amogne W., Eguale T. (2024). Compliance with infection prevention and control standard precautions and factors associated with noncompliance among healthcare workers working in public hospitals in Addis-Ababa. Antimicrob. Resist. Infect. Control.

[21] Eze P., Idemili J.C., Nwoko F.O., James N., Lawani L.O. (2024). Epidemic preparedness and response capacity against infectious disease outbreaks in 186 countries, 2018–2022. BMC Infect. Dis..

[22] Omisakin F.D. (2018). Nurses’ practices towards prevention and control of nosocomial infections in Madonna University Teaching Hospital Elele Rivers State. Nurs. Primary Care.

[23] Bhebhe L.T., Van Rooyen C., Steinberg W.J. (2014). Attitudes, knowledge and practices of healthcare workers regarding occupational exposure of pulmonary tuberculosis. Afr. J. Prim. Health Care Fam. Med..

[24] Yazie T.D., Sharew G.B., Abebe W. (2019). Knowledge, attitude, and practice of healthcare professionals regarding infection prevention at Gondar University referral hospital, northwest Ethiopia: A cross-sectional study. BMC Res. Notes.

[25] Desta M., Ayenew T., Sitotaw N., Tegegne N., Dires M., Getie M. (2018). Knowledge, practice and associated factors of infection prevention among healthcare workers in Debre Markos referral hospital, Northwest Ethiopia. BMC Health Serv. Res..

[26] Hussein S., Estifanos W.M., Melese E.S., Moga F.E. (2017). Knowledge, attitude and practice of infection prevention measures among health care workers in Wolaitta Sodo Otona teaching and referral hospital. J. Nurs. Care.

[27] Kabir A.A., Akhter F., Sharmin M., Akhter K., Begum M.B., Saha A.K., Ahmed I. (2018). Knowledge, attitude and practice of staff nurses on hospital acquired infections in tertiary care hospital of Dhaka city. North Int. Med. Coll..

[28] Ogoina D., Pondei K., Adetunji B., Chima G., Isichei C., Gidado S. (2015). Knowledge, attitude and practice of standard precautions of infection control by hospital workers in two tertiary hospitals in Nigeria. J. Infect. Prev..

[29] Gebresilassie A., Kumei A., Yemane D. (2014). Standard precautions practice among health care workers in public health facilities of Mekelle special zone, Northern Ethiopia. J. Community Med. Health Educ..

[30] Yosef T., Asefa A., Amsalu H., Alie M.S., Habte A., Ashuro Z., Tesfaw A., Shifera N. (2025). Occupational exposure to needle stick and sharp injuries and postexposure prophylaxis utilization among healthcare professionals in Southwest Ethiopia. Can. J. Infect. Dis. Med. Microbiol..

[31] Laher A.E., McDowall J. (2019). Cross-sectional survey on occupational needle stick injuries amongst prehospital emergency medical service personnel in Johannesburg. Afr. J. Emerg. Med..

[32] Matsubara C., Sakisaka K., Sychreun V., Phensavanh A., Ali M. (2020). Anxiety and psychological impact associated with needle stick and sharp device injury among tertiary workers, Vientiane, Lao PDR. Ind. Health.

