# Supplementary material for: Occupational Infection Prevention Among Nurses and Laboratory Technicians Amidst Multiple Health Emergencies in Outbreak-Prone Country, D.R. Congo
Source: Trop Med Infect Dis. 2026 Jan 2;11(1):14. doi: 10.3390/tropicalmed11010014 (PMC12845926; doi:10.3390/tropicalmed11010014)
Supplement: Supplementary file 1 [file tropicalmed-11-00014-s001.zip › tropicalmed-3979619-supplementary.pdf]

Code:

QUESTIONNAIRE

"Congo Healthcare Workers' Occupational Safety and Risk of Blood ／ bodyfluid-borne Infections in context of Complex Emergencies"

I. IDENTITY

a. Age (y)	[1] 18 – 30	[2] 31 – 40	[3] 41 – 50	[4] 51 – 60	[5] 61 or more
b. Gender	[1] F	[2] M			
c. Occupation	[1] Nurse A2	[2] Nurse A1	[3] Nurse A0	[4] Lab Technician	
d. Unit/service/ department	[1] surgery	[2] Internal Medecine	[3] Gyneco-obstetrics		
[4] Anesthesiology	[5] Medic. Lab	[6] Operation room	[7] Other		
e. Working years	[1] 1 – 5	[2] 6 – 10	[3] 11 – 15	[4] 16 or more	

II. TRAINING & KNOWLEDGE ON STANDARD PRECAUTIONS

1. Have you ever joined a training in relation to the risk of blood/body fluid-borne infection ?

[1] No	[2] Yes

2. Have you ever joined a training on standards precautions for prevention of blood/body fluid-borne infections in healthcare workers ?

[1] No	[2] Yes

3. Are there posters or other awareness materials on universal precautions for prevention of accidental blood/body exposure in your service/unit ?

[1] No	[2] Yes

III. ACCIDENTAL EXPOSURE TO BLOOD/BODY FLUID (NSI, SI, BBF)

4. In your career, how many times have you sustained accidental contact with blood/body fluid following a needle-stick, sharp injury or simple skin contact (BBF splash)?

[1] Never	[2] 1 - 5 times	[3] 6 - 10 times	[4] 11 or more

5. In the previous 12 months, have you sustained accidental contact with blood/body fluid f(BBF) following a needle-stick, sharp injury or simple skin contact (BBF splash)?

a. Injury by needle-stick ?	[1] No	[2] Yes
b. Wound/cut by sharp device ?	[1] No	[2] Yes
c. by BBF splash?	[1] No	[2] Yes

(Notes. *Questions 11, 12, 13 , 14 , 15 and 16 for those who have been exposed to blood/body fluid at least once in the previous 12 months*)

6. If yes, how many times ?

[1] 1 - 5 times	[2] 6 - 10 times	[3] 11 times or more

7. During which circumstance (procedure, activity …) did the injuries/exposure to blood/body fluid occur? (you may choose 1 or more answers :

[1] injection(IM/IV)	[2] transfusion	[3] suture	[4] wound care
[5] infiltration	[6] ponction	[7] perfusion	[8] biopsy
[9] catheterisation	[10] surgery	[11] delivery assistance	[12] other

8. Place of occurrence of accidental injury/exposure to blood or other body fluid (BBF) :

[1] Patient room	[2] Emergency unit/room	[3] Examination room	[4] corridor
[5] Care room	[6] Delivery room	[7] Devices cleaning room	[8] other

9. Timing of occurrence of accidental injuries or exposures to blood/body fluid :

[1] Start of work (day time)	[2] End of work	[3] During night work (night shift)

10. If Yes, what was your first reaction ?

[1] Causing a bleeding	[2] washing with water + soap	[3] use of disinfectant
[4] Wipe with a towel/handkerchief	[5] Other.	

11. If Yes, did you declare it to competent authority in your hospital for a post-exposure follow-up/care ?

[1] No	[2] Yes

12. Did you undergo post-exposure HIV, Hepatitis B & C testing?

[1] No	[2] Yes

13. If Yes, what were the results :

❖ 13a.HIV/AIDS:	[1] Negative	[2] Positive
❖ 13b. HEPATITIS B :	[1] Negative	[2] Positive

❖ 13c. HEPATITIS C : [1] Negative [2] Positive

14. What was the result of serological test in the source patient?

[1] Negative	[2] Positive	[3] Unknown

IV. PREVENTIVE MEASURES IN THE HOSPITAL

15. Do you always use gloves before performing any medical procedure or manipulation exposing to blood or other body fluids ?

[1] No	[2] Yes

16. Do you always use wear mask and goggles during procedures/manipulations exposing to blood/body fluid splash ?

[1] No	[2] **Yes**, always	[3] Yes, sometimes

17. Do you always wash hands with soap and then disinfect them before and after every medical/care procedure ?

[1] No	[2] **Yes**, always	[3] Yes, sometimes

18. Do you recap syringes, catheter needles after use ?

[1] No	[2] **Yes,** always	[3] Yes, sometimes

19. In your service/department, do you use 'Safety engineered (medical) devices (serynges, catheters...) and special boxes for disposure of sharp devices and needles ?

[1] No	[2] Yes

20. Do you always desinfect medical devices/tools and unclean surfaces after use ?

[1] No	[2] **Yes**, always	[3] Yes, sometimes

21. Have been vaccinated against :

❖ 21a. TETANUS : [1] No [2] Yes
❖ 21b. HEPATITIS B : [1] No [2] Yes

❖ 21c. HEPATITIS C : [1] No [2] Yes

22. Do you feel protected against the risk of accidental exposure to blood and other body fluids in your current working conditions?

[1] No	[2] Yes

23. Have you worked as Outbreak Response Team member in an area affected by the following epidemics (within the country):

❖ 23a. EBOLA ?: [1] No [2] Yes
❖ 23b. CHOLERA ?: [1] No [2] Yes
❖ 23c. YELLOW FEVER ?: [1] No [2] Yes
❖ 23d. CHIKUNGUNYA ?: [1] No [2] Yes

V. POST-EXPOSURE PROPHYLAXIS/CARE, IMPACT ON MENTAL HEALTH

24. Is the medical and psycho-social post-exposure prophylaxis/care organized in your workplace/hospital?

[1] No	[2] Yes

25. If you have been exposed to blood or other bodily fluid through needle, sharp device or other medical device during working time :

(1) 25a. After exposure to blood/body fluid of a patient, did you feel nervous or anxious?

[1] No	[2] Yes

(2) 25b. Did you feel abandoned ?

[1] No	[2] Yes

(3) 25c. Did you see a doctor or other healthcare professional ?

[1] Non	[2] Oui

THANKS FOR YOUR COLLABORATION !
